# Supplementary material for: Reducing the Kidney Uptake of High Contrast CXCR4 PET Imaging Agents via Linker Modifications
Source: Pharmaceutics. 2022 Jul 20;14(7):1502. doi: 10.3390/pharmaceutics14071502 (PMC9316317; doi:10.3390/pharmaceutics14071502)
Supplement: Supplementary file 1 [file pharmaceutics-14-01502-s001.zip › pharmaceutics-1783229-supplementary.pdf]

## Methods

### Chemical Synthesis

Unless otherwise noted, amino acid couplings were performed using 4/8/4 equivalents of the Fmoc-Amino Acid/DIC/Oxyma for 6 mins at 90ºC using the CEM Liberty Blue Microwave Peptide Synthesizer. Fmoc groups were removed after amino acid couplings were completed using a 20% piperidine solution in DMF for 1 min at 90ºC unless otherwise noted. The resin was washed three times with 3 mL DMF after each deprotection. Peptides were deprotected and simultaneously cleaved from the resin using a 92.5/5/2.5 TFA/TIS/$H_2O$ cocktail unless otherwise stated.

*Synthesis of BL06*

Fmoc-Rink Amide ProTide resin (CEM, 0.25 mmol, 0.58 mmol/g) was deprotected with 20% v/v piperidine in DMF for 1 min at 90ºC twice. Fmoc-Lys(ivDde)-OH was then coupled to the resin. The resin was then capped using 1-acetylimidazole in DMF (0.1 w/v) at room temperature for 30 minutes. Fmoc-Lys(iPr,Boc)-OH, Fmoc-D-Glu(OAll)-OH, Fmoc-Gly-OH (two coupling cycles), Fmoc-2Nal-OH (two coupling cycles), Fmoc-D-Arg-OH (two coupling cycles for 4 mins each), Fmoc-Lys(iPr,Boc)-OH, Fmoc-Tyr(tBu)-OH, and Fmoc-Phe-OH (two coupling cycles) were sequentially coupled to the peptidyl resin. At a 0.1 mmol scale, the -OAllyl protecting group on D-Glu was removed using $Pd(PPh_3)_4$ (25 mg)/Phenylsilane (700 µL) in DCM (5 mL) (2 × 6 min at 35$^o$C), with 3 washes of 3 mL of DCM in between deprotections. The $N^\alpha$-Fmoc on Phe was then removed, and cyclization was performed using DIC/HOBt in DMF (3 × 10 min at 90$^o$C). Following cyclization, the ivDde protecting group was removed by 2% v/v hydrazine in DMF (5 × 5 min at RT). The resin (0.025 mmol) containing the macrocyclic peptide was

coupled with Fmoc-Pip-OH/HATU/DIEA in DMF for 10 min at 50ºC for two cycles. The chelator DOTA tri-*t*-butyl ester (4 equiv.) in DMF was coupled to the terminal amine with HATU/DIEA (4/8 equiv.) for 10 minutes at 50ºC, with two coupling cycles. The peptide was deprotected and cleaved for 4 h at 35 ºC and the crude peptide mixture was worked up as previously described. The reaction mixture was purified by HPLC using the preparative column eluted with 10-25% acetonitrile in water with 0.1% TFA for 0-15 mins at a flow rate of 30 mL/min. The retention time was 14.0 min. ESI-MS: calculated $[M+2H]^{2+}$ for BL06 $C_{91}H_{140}N_{22}O_{19}$ 922.53; found $[M+2H]^{2+}$ 922.89.

*Synthesis of Ga-BL06*

For Ga-BL06, a solution of BL06 (1.54 mg, 0.84 µmol) and $GaCl_3$ (29.3 µL, 0.2 M, 5.85 µmol) in 500 µL sodium acetate buffer (0.1 M, pH 4.2) was incubated at 80°C for 15 min. The reaction mixture was purified by HPLC using the preparative column eluted with 10-25% acetonitrile in water with 0.1% TFA for 0-15 min at a flow rate of 30 mL/min. The retention time of Ga-BL06 was 13.6 min, and the yield of the peptide was 87%. ESI-MS: calculated $[M+2H]^{2+}$ for Ga-BL06 $C_{91}H_{138}GaN_{22}O_{19}$ 955.99; found $[M+2H]^{2+}$ 956.86.

*Synthesis of BL20*

From the synthesis of BL06, following the removal of the ivDde group, the resin (0.025 mmol) was coupled with three Fmoc-D-Glu(OtBu)-OH sequentially. Afterwards, the chelator DOTA tri-t-butyl ester (4 equiv.) in DMF was coupled to the terminal amine with HATU/DIEA (4/8 equiv.) for 18 hours at room temperature. The peptide was deprotected and cleaved for 3.5 h at 35 °C and the crude peptide mixture was worked up

as previously described. The reaction mixture was purified by HPLC using the preparative column eluted with 11-31% acetonitrile in water with 0.1% TFA over 20 mins at a flow rate of 30 mL/min. The retention time was 13.3 min. ESI-MS: calculated [M+2H]2+ for BL20 $C_{99}H_{147}N_{23}O_{27}$ 1046.55; found [M+2H]2+ 1045.91.

*Synthesis of Ga-BL20*

For Ga-BL20, a solution of BL20 (0.94 mg, 0.45 μmol) and GaCl3 (13 μL, 0.2 M, 2.6 μmol) in 500 μL sodium acetate buffer (0.1 M, pH 4.2) was incubated at 80°C for 15 min. The reaction mixture was purified by HPLC using the preparative column eluted with 11-31% acetonitrile in water with 0.1% TFA for 30 min at a flow rate of 30 mL/min. The retention time of Ga-BL20 was 13.4 min. ESI-MS: calculated [M+2H]2+ for Ga-BL20 $C_{99}H_{147}GaN_{23}O_{27}$ 1080.01; found [M+2H]2+ 1079.34.

*Synthesis of BL17*

From the synthesis of BL06, following the removal of the ivDde group, the resin (0.025 mmol) was coupled with three Fmoc-Aad(OtBu)-OH sequentially. Afterwards, the chelator DOTA tri-*t*-butyl ester (4 equiv.) in DMF was coupled to the terminal amine with HATU/DIEA (4/8 equiv.) for 18 hours at room temperature. The peptide was deprotected and cleaved for 3.5 h at 35 $^{o}$C and the crude peptide mixture was worked up as previously described. The reaction mixture was purified by HPLC using the preparative column eluted with 10-30% acetonitrile in water with 0.1% TFA over 20 mins at a flow rate of 30

mL/min. The retention time was 14.3 min. ESI-MS: calculated $[M+2H]^{2+}$ for BL17 $C_{102}H_{155}N_{23}O_{27}$ 1067.57; found $[M+2H]^{2+}$ 1067.41.

*Synthesis of Ga-BL17*

For Ga-BL17, a solution of BL17 (2.3 mg, 1.1 µmol) and $GaCl_3$ (27 µL, 0.2 M, 5.4 µmol) in 500 µL sodium acetate buffer (0.1 M, pH 4.2) was incubated at 90°C for 20 min. The reaction mixture was purified by HPLC using the preparative column eluted with 10-30% acetonitrile in water with 0.1% TFA over 20 mins at a flow rate of 30 mL/min. The retention time of Ga-BL17 was 14.6 min, and the yield of the peptide was 86%. ESI-MS: calculated $[M+2H]^{2+}$ for Ga-BL17 $C_{102}H_{153}GaN_{23}O_{27}$ 1101.03; found $[M+2H]^{2+}$ 1100.98.

*Synthesis of BL25*

From the synthesis of BL06, following the removal of the ivDde group, the resin (0.025 mmol) was coupled with three Fmoc-D-Asp(OBno)-OH sequentially using a 2/4/2 equiv. of amino acid/DIC/Oxyma. The Fmoc was deprotected at room temperature for 5 minutes between couplings. Afterwards, the chelator DOTA tri-*t*-butyl ester (4 equiv.) in DMF was coupled to the terminal amine with HATU/DIEA (4/8 equiv.) for 18 hours at room temperature. The peptide was deprotected and cleaved for 3.5 h at 35 $^{o}$C and the crude peptide mixture was worked up as previously described. The reaction mixture was purified by HPLC using the preparative column eluted with 12-32% acetonitrile in water with 0.1% TFA for 0-20 min at a flow rate of 30 mL/min. The retention time was 12.0 min. ESI-MS: calculated $[M+2H]^{2+}$ for BL25 $C_{96}H_{143}N_{23}O_{27}$ 1025.53; found $[M+2H]^{2+}$ 1024.95.

*Synthesis of Ga-BL25*

For Ga-BL25, a solution of BL25 (2.06 mg, 1.0 μmol) and $GaCl_3$ (27.8 μL, 0.2 M, 5.55 μmol) in 500 μL sodium acetate buffer (0.1 M, pH 4.2) was incubated at 80°C for 15 min. The reaction mixture was purified by HPLC using the preparative column eluted with 12-32% acetonitrile in water with 0.1% TFA for 0-20 min at a flow rate of 30 mL/min. The retention time was 12.3 min, and the yield of the peptide was 76%. ESI-MS: calculated $[M+3H]^{3+}$ for Ga-BL25 $C_{96}H_{143}GaN_{23}O_{27}$ 706.66; found $[M+3H]^{3+}$ 706.20.

*Synthesis of BL31*

From the synthesis of BL06, following the removal of the ivDde group, the resin (0.025 mmol) was coupled with three Fmoc-CysAcid-OH sequentially. Afterwards, the chelator DOTA tri-t-butyl ester (4 equiv.) in DMF was coupled to the terminal amine with HATU/DIEA (4/8 equiv.) for 18 hours at room temperature. The peptide was deprotected and cleaved for 3.5 h at 35 °C and the crude peptide mixture was worked up as previously described. The reaction mixture was purified by HPLC using the preparative column eluted with 11-31% acetonitrile in water with 0.1% TFA over 20 mins at a flow rate of 30 mL/min. The retention time was 13.0 min. ESI-MS: calculated $[M+2H]^{2+}$ for BL31 $C_{93}H_{143}N_{23}O_{30}S_3$ 1079.48; found $[M+2H]^{2+}$ 1079.56.

*Synthesis of Ga-BL31*

For Ga-BL31, a solution of BL31 (1.34 mg, 0.62 μmol) and GaCl3 (15.5 μL, 0.2 M, 3.1 μmol) in 500 μL sodium acetate buffer (0.1 M, pH 4.2) was incubated at 80°C for 15 min. The reaction mixture was purified by HPLC using the preparative column eluted with

11-31% acetonitrile in water with 0.1% TFA for 30 min at a flow rate of 30 mL/min. The retention time of Ga-BL31 was 11.7 min. ESI-MS: calculated $[M+2H]^{2+}$ for Ga-BL31 $C_{93}H_{141}GaN_{23}O_{30}S_3$ 1112.93; found $[M+2H]^{2+}$ 1112.21.

*Synthesis of BL30*

From the synthesis of BL06, following the removal of the ivDde group, the resin (0.025 mmol) was coupled with three Fmoc-Glu(OtBu)-OH sequentially. Afterwards, the chelator DOTAGA tetra-t-butyl ester (4 equiv.) in DMF was coupled to the terminal amine with HATU/DIEA (4/8 equiv.) for 18 hours at room temperature. The peptide was deprotected and cleaved for 3.5 h at 35°C and the crude peptide mixture was worked up as previously described. The reaction mixture was purified by HPLC using the preparative column eluted with 11-31% acetonitrile in water with 0.1% TFA over 20 mins at a flow rate of 30 mL/min. The retention time was 12.98 min. ESI-MS: calculated $[M+2H]^{2+}$ for BL30 $C_{102}H_{153}N_{23}O_{29}$ 1082.56; found $[M+2H]^{2+}$ 1082.89.

*Synthesis of Ga-BL30*

For Ga-BL30, a solution of BL30 (1.88 mg, 0.87 μmol) and GaCl3 (21.7 μL, 0.2 M, 4.34 μmol) in 500 μL sodium acetate buffer (0.1 M, pH 4.2) was incubated at 80°C for 15 min. The reaction mixture was purified by HPLC using the preparative column eluted with 11-31% acetonitrile in water with 0.1% TFA for 30 min at a flow rate of 30 mL/min. The retention time of Ga-BL30 was 11.70 min. ESI-MS: calculated $[M+2H]^{2+}$ for Ga-BL30 $C_{102}H_{150}GaN_{23}O_{29}$ 1115.51; found $[M+2H]^{2+}$ 1115.23.

**Radiochemical Data**

Method A: 4.5 mL/min, 23% ACN (0.1% TFA)

Method B: 2 mL/min, 23% ACN (0.1% TFA)

Method C: 2 mL/min, 25% ACN (0.1% TFA)

Method D: 2 mL/min, 26% ACN (0.1% TFA)

Method E: 4.5 mL/min, 24% ACN (0.1% TFA)

Method F: 2 mL/min, 24% ACN (0.1% TFA)

Method G: 2 mL/min, 27% ACN (0.1% TFA)

Method H: 4.5 mL/min, 25% ACN (0.1% TFA)

Method I: 4.5 mL/min, 16% ACN (0.1% AcOH)

Method J: 2 mL/min, 18% ACN (0.1% AcOH)

BL06 was radiolabeled with [$^{68}$Ga]$GaCl_3$ as outlined in the Methods section. The mixture was purified by semi-prep HPLC (Method A) with a retention time of 16.6 mins, and quality control was performed by analytical HPLC (Method B) with the co-injection of the unlabeled standard with a one-twelfth of the radiotracer, with a retention time of 12.2 mins.

BL17 was radiolabeled with [$^{68}$Ga]$GaCl_3$ as outlined in the Methods section. The mixture was purified by semi-prep HPLC (Method A) with a retention time of 24.8 mins, and quality control was performed by analytical HPLC (Method C) with the co-injection of the unlabeled standard with a one-twelfth of the radiotracer, with a retention time of 6.8 mins.

BL20 was radiolabeled with [$^{68}$Ga]$GaCl_3$ as outlined in the Methods section. The mixture was purified by semi-prep HPLC (Method A) with a retention time of 21.9 mins, and quality control was performed by analytical HPLC (Method D) with the co-injection of the unlabeled standard with a one-twelfth of the radiotracer, with a retention time of 6.1 mins.

BL25 was radiolabeled with [$^{68}$Ga]$GaCl_3$ as outlined in the Methods section. The mixture was purified by semi-prep HPLC (Method E) with a retention time of 16.2 mins, and quality control was performed by analytical HPLC (Method F) with the co-injection of the unlabeled standard with a one-twelfth of the radiotracer, with a retention time of 5.2 mins.

BL30 was radiolabeled with [$^{68}$Ga]$GaCl_3$ as outlined in the Methods section. The mixture was purified by semi-prep HPLC (Method A) with a retention time of 25.3 mins, and quality control was performed by analytical HPLC (Method G) with the co-injection of the unlabeled standard with a one-twelfth of the radiotracer, with a retention time of 5.7 mins.

BL31 was radiolabeled with [$^{68}$Ga]$GaCl_3$ as outlined in the Methods section. The mixture was purified by semi-prep HPLC (Method H) with a retention time of 25.3 mins, and quality control was performed by analytical HPLC (Method G) with the co-injection of the unlabeled standard with a one-twelfth of the radiotracer, with a retention time of 7.1 mins.

Pentixafor was radiolabeled with [$^{68}$Ga]$GaCl_3$ as outlined in the Methods section. The mixture was purified by semi-prep HPLC (Method I), with a retention time of 31.9

mins, and quality control was performed by analytical HPLC 2 mL/min, (Method J) with the co-injection of the unlabeled standard with a one-twelfth of the radiotracer, with a retention time of 7.5 mins.

## Imaging Data

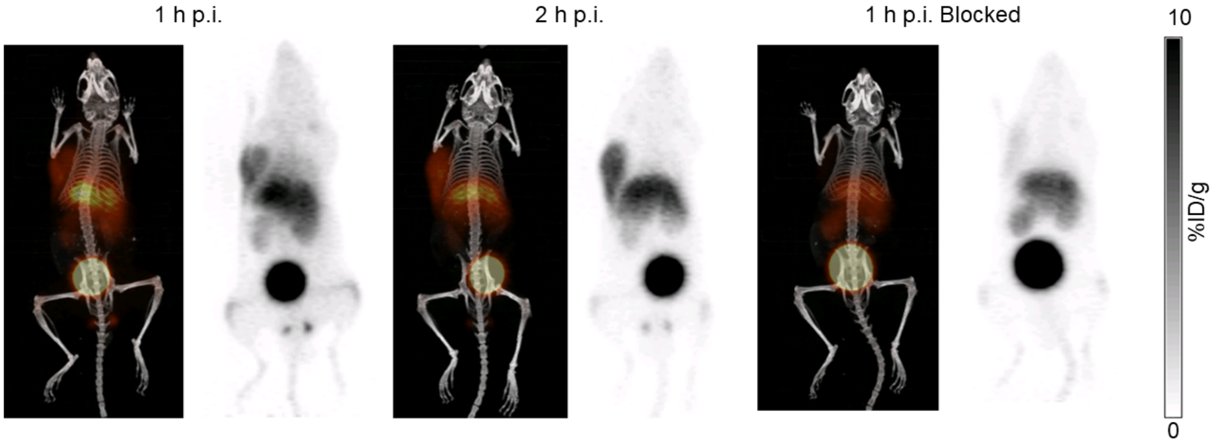


**Supplementary Figure S1.** Maximum intensity projections for PET/CT and PET alone at 1 h p.i., 2 h p.i. and 1 h p.i. blocked of [$^{68}$Ga]Ga-BL06. Blocking was performed via i.p. injection of 7.5 μg of LY2510924 15 mins prior. Scales of the PET images is 0-10 %ID/g.

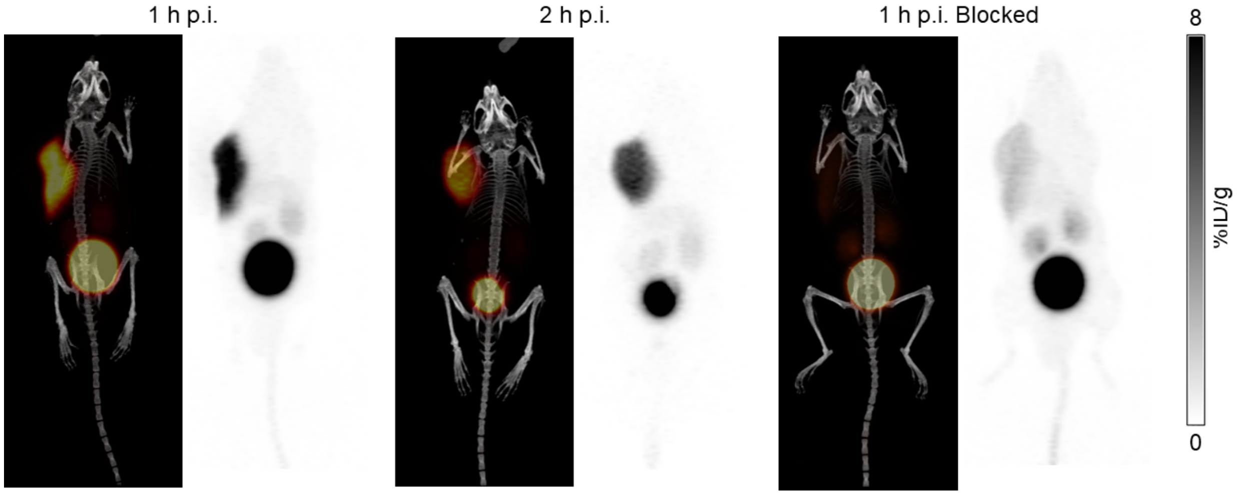


**Supplementary Figure S2.** Maximum intensity projections for PET/CT and PET alone at 1 h p.i., 2 h p.i. and 1 h p.i. blocked of [$^{68}$Ga]Ga-BL31. Blocking was performed via i.p. injection of 7.5 μg of LY2510924 15 mins prior. Scales of the PET images are 0-8 %ID/g.

**Biodistribution Data**

**Supplementary Table S1.** Biodistribution data (%ID/g) of [$^{68}$Ga]Ga-Pentixafor in Daudi tumor-bearing mice at selected time points. Mice in the 1 h blocked group received an injection of 7.5 µg of LY2510924 (i.p.) 15 min before tracer administration.

| Radiotracer | Molar Activity (GBq/µmol) | Radiochemical Purity (%) | Radiochemical Yield (%) |
|---|---|---|---|
| **[$^{68}$Ga]BL02** | 258±90$^{**}$ | >99%$^{**}$ | 64.0±9.9%$^{**}$ |
| **[$^{68}$Ga]BL06** | 54±36$^{x}$ | >99%$^{x}$ | 63.8±2.6$^{x}$ |
| **[$^{68}$Ga]BL17** | N.D | >97%$^{x}$ | 64.6±1.6$^{x}$ |
| **[$^{68}$Ga]BL20** | 98.79$^{*}$ | >99% | 70.5$^{*}$ |
| **[$^{68}$Ga]BL25** | N.D | >99%$^{*}$ | 61.7$^{*}$ |
| **[$^{68}$Ga]BL30** | 81.77$^{*}$ | >99%$^{*}$ | 75.0$^{*}$ |
| **[$^{68}$Ga]BL31** | 429±180$^{†}$ | >99%$^{†}$ | 63.8±2.6$^{†}$ |

$^{*}$ n = 1, $^{x}$ n = 2,$^{†}$ n = 4, $^{**}$ n = 9, N.D: not determined.

**Supplementary Table S2.** Biodistribution data (%ID/g) of [$^{68}$Ga]Ga-BL06 in Daudi tumor-bearing mice at selected time points. Mice in the 1 h blocked group received an injection of 7.5 µg of LY2510924 (i.p.) 15 min before tracer administration.

| **[$^{68}$Ga]Ga-BL06** | **1 h** | | | **1 h blocked** | | | **2 h** | | |
|---|---|---|---|---|---|---|---|---|---|
| | **Mean** | **S.Dev** | **n** | **Mean** | **S.Dev** | **n** | **Mean** | **S.Dev** | **n** |
| **Blood** | 2.78 | 0.46 | 6 | 1.17 | 0.26 | 7 | 1.11 | 0.24 | 8 |
| **Fat** | 0.35 | 0.10 | 6 | 0.16 | 0.09 | 7 | 0.19 | 0.04 | 8 |
| **Testes** | 0.61 | 0.10 | 6 | 0.34 | 0.06 | 7 | 0.46 | 0.02 | 8 |
| **Intestine** | 1.07 | 0.13 | 6 | 0.70 | 0.18 | 7 | 0.68 | 0.11 | 8 |
| **Stomach** | 0.29 | 0.08 | 6 | 0.14 | 0.04 | 7 | 0.21 | 0.07 | 8 |
| **Spleen** | 15.53 | 1.83 | 6 | 3.30 | 0.73 | 7 | 9.14 | 1.60 | 8 |
| **Liver** | 8.60 | 0.77 | 6 | 9.25 | 0.94 | 6 | 10.35 | 0.40 | 8 |
| **Pancreas** | 0.70 | 0.09 | 6 | 0.29 | 0.08 | 7 | 0.39 | 0.07 | 7 |
| **Adrenals** | 3.61 | 1.14 | 5 | 0.80 | 0.33 | 7 | 3.19 | 1.11 | 8 |
| **Kidney** | 6.25 | 1.05 | 6 | 8.26 | 2.79 | 7 | 4.82 | 0.61 | 8 |
| **Lung** | 15.00 | 2.06 | 6 | 2.20 | 0.55 | 7 | 7.18 | 0.86 | 8 |
| **Heart** | 1.73 | 0.25 | 6 | 0.48 | 0.11 | 7 | 0.93 | 0.17 | 8 |
| **Muscle** | 0.41 | 0.11 | 6 | 0.19 | 0.04 | 7 | 0.21 | 0.06 | 8 |
| **Bone** | 1.84 | 0.65 | 6 | 0.82 | 0.26 | 7 | 1.11 | 0.25 | 8 |
| **Brain** | 0.06 | 0.00 | 6 | 0.03 | 0.01 | 7 | 0.03 | 0.00 | 8 |
| **Tumor** | 10.26 | 1.29 | 6 | 2.06 | 0.62 | 7 | 11.32 | 1.44 | 8 |
| | | | | | | | | | |
| **Ratios** | | | | | | | | | |
| **Tumor:Blood** | 3.75 | 1.18 | 6 | 1.84 | 0.74 | 7 | 10.35 | 1.33 | 8 |
| **Tumor:Liver** | 1.19 | 0.56 | 6 | 0.21 | 0.05 | 6 | 1.10 | 0.14 | 8 |
| **Tumor:Spleen** | 0.67 | 0.17 | 6 | 0.64 | 0.19 | 7 | 1.25 | 0.13 | 8 |
| **Tumor:Muscle** | 25.59 | 4.15 | 6 | 10.97 | 2.86 | 7 | 57.98 | 13.51 | 8 |
| **Tumor:Bone** | 6.04 | 1.30 | 6 | 2.77 | 1.10 | 7 | 10.56 | 2.36 | 8 |
| **Tumor:Lungs** | 0.69 | 0.21 | 6 | 0.95 | 0.26 | 7 | 1.59 | 0.21 | 8 |

**Supplementary Table S3.** Biodistribution data (%ID/g) of [$^{68}$Ga]Ga-BL20 in Daudi tumor-bearing mice at selected time points. Mice in the 1 h blocked group received an injection of 7.5 µg of LY2510924 (i.p.) 15 min before tracer administration.

| **[$^{68}$Ga]Ga-BL20** | **1 h** | | | **1 h blocked** | | | **2 h** | | |
|---|---|---|---|---|---|---|---|---|---|
| | **Mean** | **S.Dev** | **n** | **Mean** | **S.Dev** | **n** | **Mean** | **S.Dev** | **n** |
| **Blood** | 0.42 | 0.12 | 3 | 0.65 | na | 1 | 0.08 | 0.02 | 4 |
| **Fat** | 0.06 | 0.03 | 3 | 0.12 | na | 1 | 0.01 | 0.01 | 4 |
| **Testes** | 0.15 | 0.03 | 3 | 0.20 | na | 1 | 0.05 | 0.01 | 4 |
| **Intestine** | 0.21 | 0.02 | 3 | 0.37 | na | 1 | 0.25 | 0.25 | 4 |
| **Stomach** | 0.05 | 0.01 | 3 | 0.24 | na | 1 | 0.05 | 0.03 | 4 |
| **Spleen** | 0.49 | 0.07 | 3 | 0.30 | na | 1 | 0.21 | 0.06 | 4 |
| **Liver** | 0.58 | 0.07 | 3 | 0.44 | na | 1 | 0.51 | 0.04 | 4 |
| **Pancreas** | 0.11 | 0.03 | 3 | 0.16 | na | 1 | 0.04 | 0.01 | 4 |
| **Adrenals** | 0.35 | 0.20 | 3 | 0.35 | na | 1 | 0.27 | 0.17 | 4 |
| **Kidney** | 3.58 | 0.26 | 3 | 5.92 | na | 1 | 3.02 | 0.22 | 4 |
| **Lung** | 0.51 | 0.08 | 3 | 0.71 | na | 1 | 0.22 | 0.04 | 4 |
| **Heart** | 0.15 | 0.03 | 3 | 0.22 | na | 1 | 0.05 | 0.01 | 4 |
| **Muscle** | 0.08 | 0.02 | 3 | 0.13 | na | 1 | 0.02 | 0.00 | 4 |
| **Bone** | 0.17 | 0.00 | 3 | 0.15 | na | 1 | 0.05 | 0.03 | 4 |
| **Brain** | 0.01 | 0.00 | 3 | 0.02 | na | 1 | 0.01 | 0.00 | 4 |
| **Tumor** | 9.07 | 0.76 | 3 | 0.59 | na | 1 | 8.01 | 1.39 | 4 |
| | | | | | | | | | |
| **Ratios** | | | | | | | | | |
| **Tumour:Blood** | 22.78 | 7.35 | 3 | 0.90 | na | 1 | 113.17 | 45.03 | 4 |
| **Tumour:Liver** | 15.68 | 0.90 | 3 | 1.34 | na | 1 | 15.57 | 1.73 | 4 |
| **Tumour:Spleen** | 18.55 | 1.38 | 3 | 1.96 | na | 1 | 41.58 | 13.90 | 4 |
| **Tumour:Muscle** | 125.40 | 54.59 | 3 | 4.49 | na | 1 | 346.93 | 81.84 | 4 |
| **Tumour:Bone** | 54.57 | 6.00 | 3 | 3.88 | na | 1 | 189.39 | 109.18 | 4 |
| **Tumour:Lung** | 18.12 | 3.56 | 3 | 0.82 | na | 1 | 37.40 | 8.71 | 4 |

**Supplementary Table S4.** Biodistribution data (%ID/g) of [$^{68}$Ga]Ga-BL17 in Daudi tumor-bearing mice at selected time points. Mice in the 1 h blocked group received an injection of 7.5 µg of LY2510924 (i.p.) 15 min before tracer administration.

| [$^{68}$Ga]Ga-BL17 | 1 h | | | 1 h blocked | | | 2 h | | |
|---|---|---|---|---|---|---|---|---|---|
| | **Mean** | **S.Dev** | **n** | **Mean** | **S.Dev** | **n** | **Mean** | **S.Dev** | **n** |
| **Blood** | 0.41 | 0.12 | 4 | 1.40 | 0.14 | 2 | 0.09 | 0.01 | 4 |
| **Fat** | 0.04 | 0.01 | 4 | 0.32 | 0.01 | 2 | 0.02 | 0.01 | 4 |
| **Testes** | 0.20 | 0.05 | 4 | 0.53 | 0.16 | 2 | 0.06 | 0.01 | 4 |
| **Intestine** | 0.21 | 0.07 | 4 | 0.65 | 0.08 | 2 | 0.15 | 0.07 | 4 |
| **Stomach** | 0.05 | 0.03 | 4 | 0.18 | 0.02 | 2 | 0.03 | 0.02 | 4 |
| **Spleen** | 0.33 | 0.07 | 4 | 0.81 | 0.20 | 2 | 0.30 | 0.05 | 4 |
| **Liver** | 0.50 | 0.07 | 4 | 0.84 | 0.13 | 2 | 0.47 | 0.08 | 4 |
| **Pancreas** | 0.10 | 0.03 | 4 | 0.38 | 0.05 | 2 | 0.04 | 0.02 | 4 |
| **Adrenals** | 0.16 | 0.07 | 4 | 0.79 | 0.25 | 2 | 0.20 | 0.12 | 4 |
| **Kidney** | 3.30 | 0.41 | 4 | 15.17 | 8.21 | 2 | 3.28 | 0.34 | 4 |
| **Lung** | 0.51 | 0.12 | 4 | 1.35 | 0.20 | 2 | 0.25 | 0.03 | 4 |
| **Heart** | 0.14 | 0.04 | 4 | 0.45 | 0.05 | 2 | 0.06 | 0.01 | 4 |
| **Muscle** | 0.10 | 0.03 | 4 | 0.25 | 0.00 | 2 | 0.03 | 0.01 | 4 |
| **Bone** | 0.09 | 0.03 | 4 | 0.31 | 0.05 | 2 | 0.09 | 0.03 | 4 |
| **Brain** | 0.02 | 0.01 | 4 | 0.03 | 0.00 | 2 | 0.01 | 0.00 | 4 |
| **Tumor** | **6.32** | **0.67** | 4 | 0.68 | 0.00 | 2 | 6.40 | 1.60 | 4 |
| | | | | | | | | | |
| **Ratios** | | | | | | | | | |
| **Tumour:Blood** | 16.94 | 6.57 | 4 | 0.49 | 0.05 | 2 | 69.33 | 18.61 | 4 |
| **Tumour:Liver** | 12.66 | 0.79 | 4 | 0.81 | 0.13 | 2 | 13.69 | 4.46 | 4 |
| **Tumour:Spleen** | 19.76 | 3.57 | 4 | 0.87 | 0.22 | 2 | 21.11 | 6.42 | 4 |
| **Tumour:Muscle** | 67.93 | 16.71 | 4 | 2.74 | 0.05 | 2 | 256.87 | 72.49 | 4 |
| **Tumour:Bone** | 76.60 | 21.25 | 4 | 2.24 | 0.35 | 2 | 79.15 | 32.79 | 4 |
| **Tumour:Lung** | 12.64 | 2.48 | 4 | 0.51 | 0.07 | 2 | 25.37 | 6.19 | 4 |

**Supplementary Table S5.** Biodistribution data (%ID/g) of [$^{68}$Ga]Ga-BL25 in Daudi tumor-bearing mice at selected time points.

| **[$^{68}$Ga]Ga-BL25** | **2 h** | | |
|---|---|---|---|
| | **Mean** | **S.Dev** | **n** |
| **Blood** | 0.18 | 0.01 | 4 |
| **Fat** | 0.02 | 0.01 | 4 |
| **Testes** | 0.07 | 0.00 | 4 |
| **Intestine** | 0.13 | 0.03 | 4 |
| **Stomach** | 0.03 | 0.02 | 4 |
| **Spleen** | 0.19 | 0.03 | 4 |
| **Liver** | 0.47 | 0.04 | 4 |
| **Pancreas** | 0.05 | 0.01 | 4 |
| **Adrenals** | 0.10 | 0.01 | 4 |
| **Kidney** | 1.92 | 0.07 | 4 |
| **Lung** | 0.37 | 0.11 | 4 |
| **Heart** | 0.07 | 0.00 | 4 |
| **Muscle** | 0.04 | 0.00 | 4 |
| **Bone** | 0.06 | 0.02 | 4 |
| **Brain** | 0.01 | 0.00 | 4 |
| **Tumor** | 5.53 | 0.29 | 4 |
| | | | |
| **Ratios** | | | |
| **Tumor:Blood** | 30.77 | 1.18 | 4 |
| **Tumor:Liver** | 12.05 | 0.76 | 4 |
| **Tumor:Spleen** | 30.72 | 3.73 | 4 |
| **Tumor:Muscle** | 159.64 | 19.97 | 4 |
| **Tumor:Bone** | 103.83 | 33.98 | 4 |
| **Tumor:Lung** | 16.13 | 4.21 | 4 |

**Supplementary Table S6.** Biodistribution data (%ID/g) of [$^{68}$Ga]Ga-BL31 in Daudi tumor-bearing mice at selected time points. Mice in the 1 h blocked group received an injection of 7.5 µg of LY2510924 (i.p.) 15 min before tracer administration.

| [$^{68}$Ga]Ga-BL31 | 1 h | | | 1 h blocked | | | 2 h | | |
|---|---|---|---|---|---|---|---|---|---|
| | **Mean** | **SD** | **n** | **Mean** | **SD** | **n** | **Mean** | **SD** | **n** |
| **Blood** | 0.35 | 0.07 | 7 | 0.58 | 0.21 | 7 | 0.08 | 0.04 | 7 |
| **Fat** | 0.05 | 0.02 | 7 | 0.10 | 0.04 | 7 | 0.02 | 0.01 | 7 |
| **Testes** | 0.17 | 0.06 | 7 | 0.21 | 0.07 | 7 | 0.06 | 0.01 | 7 |
| **Intestine** | 0.23 | 0.03 | 7 | 0.47 | 0.36 | 7 | 0.11 | 0.04 | 7 |
| **Stomach** | 0.06 | 0.03 | 6 | 0.11 | 0.06 | 7 | 0.04 | 0.01 | 6 |
| **Spleen** | 0.33 | 0.03 | 7 | 0.34 | 0.11 | 7 | 0.24 | 0.05 | 7 |
| **Liver** | 0.67 | 0.10 | 7 | 0.71 | 0.13 | 7 | 0.66 | 0.10 | 7 |
| **Pancreas** | 0.09 | 0.02 | 7 | 0.13 | 0.05 | 7 | 0.04 | 0.01 | 7 |
| **Adrenals** | 0.18 | 0.02 | 6 | 0.21 | 0.07 | 6 | 0.17 | 0.09 | 5 |
| **Kidney** | 2.54 | 0.26 | 7 | 3.83 | 1.08 | 7 | 2.23 | 0.21 | 7 |
| **Lung** | 0.56 | 0.09 | 7 | 0.67 | 0.21 | 7 | 0.26 | 0.05 | 7 |
| **Heart** | 0.14 | 0.03 | 7 | 0.19 | 0.07 | 7 | 0.05 | 0.01 | 7 |
| **Muscle** | 0.08 | 0.02 | 7 | 0.12 | 0.06 | 7 | 0.03 | 0.01 | 7 |
| **Bone** | 0.12 | 0.03 | 7 | 0.22 | 0.16 | 7 | 0.07 | 0.04 | 7 |
| **Brain** | 0.02 | 0.01 | 7 | 0.02 | 0.00 | 7 | 0.01 | 0.01 | 7 |
| **Tumor** | 9.41 | 1.00 | 7 | 0.94 | 0.54 | 6 | 8.97 | 1.45 | 7 |
| | | | | | | | | | |
| **Ratios** | | | | | | | | | |
| **Tumor:Blood** | 26.91 | 4.33 | 7 | 1.68 | 1.06 | 6 | 121.5 | 44.97 | 7 |
| **Tumor:Liver** | 14.23 | 1.84 | 7 | 1.34 | 0.67 | 6 | 13.73 | 2.01 | 7 |
| **Tumor:Spleen** | 28.40 | 2.93 | 7 | 3.12 | 2.13 | 6 | 39.28 | 9.47 | 7 |
| **Tumor:Muscle** | 128.8 | 29.07 | 7 | 9.40 | 7.01 | 6 | 302.4 | 92.32 | 7 |
| **Tumor:Bone** | 86.12 | 25.06 | 7 | 6.07 | 5.50 | 6 | 170.5 | 132.3 | 7 |
| **Tumor:Lung** | 17.15 | 2.85 | 7 | 1.44 | 1.02 | 6 | 35.39 | 6.92 | 7 |
| **Tumor:Kidney** | 3.73 | 0.52 | 7 | 0.26 | 0.16 | 6 | 3.95 | 0.54 | 7 |

**Supplementary Table S7.** Biodistribution data (%ID/g) of [$^{68}$Ga]Ga-BL30 in Daudi tumor-bearing mice at selected time points.

| **[$^{68}$Ga]Ga-BL30** | **1 h** | | |
|---|---|---|---|
| | **Mean** | **SD** | **n** |
| **Blood** | 0.27 | 0.08 | 4 |
| **Fat** | 0.03 | 0.01 | 4 |
| **Testes** | 0.10 | 0.01 | 4 |
| **Intestine** | 0.20 | 0.03 | 4 |
| **Stomach** | 0.06 | 0.01 | 3 |
| **Spleen** | 0.25 | 0.04 | 4 |
| **Liver** | 0.42 | 0.27 | 4 |
| **Pancreas** | 0.07 | 0.01 | 4 |
| **Adrenals** | 0.13 | 0.08 | 4 |
| **Kidney** | 8.99 | 1.56 | 4 |
| **Lung** | 0.43 | 0.11 | 4 |
| **Heart** | 0.11 | 0.03 | 4 |
| **Muscle** | 0.05 | 0.01 | 4 |
| **Bone** | 0.07 | 0.02 | 4 |
| **Brain** | 0.01 | 0.00 | 4 |
| **Tumor** | 5.76 | 0.77 | 4 |
| | | | |
| **Ratios** | | | |
| **Tumor:Blood** | 23.80 | 6.10 | 4 |
| **Tumor:Liver** | 16.43 | 9.13 | 4 |
| **Tumor:Spleen** | 24.47 | 1.74 | 4 |
| **Tumor:Muscle** | 122.74 | 20.39 | 4 |
| **Tumor:Bone** | 91.99 | 14.51 | 4 |
| **Tumor:Lung** | 14.17 | 2.89 | 4 |
| **Tumor:Kidney** | 0.68 | 0.03 | 4 |

**Supplementary Table S8.** Biodistribution data (%ID/g) of [$^{68}$Ga]Ga-Pentixafor in Daudi tumor-bearing mice at selected time points. Mice in the 1 h blocked group received an injection of 7.5 µg of LY2510924 (i.p.) 15 min before tracer administration.

| [$^{68}$Ga]Ga-Pentixafor | 1 h | | | 1 h blocked | | | 2 h | | |
|---|---|---|---|---|---|---|---|---|---|
| | **Mean** | **S.Dev** | **n** | **Mean** | **S.Dev** | **n** | **Mean** | **S.Dev** | **n** |
| **Blood** | 0.97 | 0.12 | 7 | 1.23 | 0.35 | 7 | 0.28 | 0.04 | 7 |
| **Fat** | 0.08 | 0.02 | 7 | 0.18 | 0.07 | 7 | 0.04 | 0.01 | 7 |
| **Testes** | 0.39 | 0.06 | 7 | 0.55 | 0.14 | 7 | 0.13 | 0.03 | 7 |
| **Intestine** | 0.43 | 0.07 | 7 | 0.59 | 0.25 | 7 | 0.20 | 0.05 | 7 |
| **Stomach** | 0.13 | 0.04 | 7 | 0.17 | 0.08 | 7 | 0.05 | 0.02 | 7 |
| **Spleen** | 0.63 | 0.09 | 7 | 0.59 | 0.19 | 7 | 0.33 | 0.08 | 7 |
| **Liver** | 1.23 | 0.15 | 7 | 1.25 | 0.25 | 7 | 0.89 | 0.12 | 7 |
| **Pancreas** | 0.21 | 0.03 | 7 | 0.25 | 0.08 | 6 | 0.08 | 0.02 | 7 |
| **Adrenals** | 0.34 | 0.12 | 7 | 0.38 | 0.14 | 7 | 0.18 | 0.09 | 7 |
| **Kidney** | 2.78 | 0.39 | 6 | 3.16 | 0.73 | 6 | 1.76 | 0.38 | 7 |
| **Lung** | 1.16 | 0.14 | 7 | 1.39 | 0.40 | 7 | 0.54 | 0.08 | 7 |
| **Heart** | 0.36 | 0.04 | 7 | 0.47 | 0.14 | 7 | 0.13 | 0.03 | 7 |
| **Muscle** | 0.20 | 0.04 | 7 | 0.26 | 0.08 | 7 | 0.06 | 0.02 | 7 |
| **Bone** | 0.18 | 0.04 | 7 | 0.27 | 0.14 | 7 | 0.14 | 0.05 | 7 |
| **Brain** | 0.03 | 0.01 | 7 | 0.03 | 0.01 | 7 | 0.01 | 0.00 | 7 |
| **Tumor** | 6.31 | 1.02 | 7 | 0.61 | 0.14 | 7 | 5.23 | 0.43 | 7 |
| | | | | | | | | | |
| **Ratios** | | | | | | | | | |
| **Tumor-to-Blood** | 6.53 | 0.97 | 7 | 0.53 | 0.16 | 7 | 18.94 | 2.67 | 7 |
| **Tumor-to-Muscle** | 33.25 | 9.31 | 7 | 2.55 | 1.10 | 7 | 95.44 | 36.68 | 7 |
| **Tumor-to-Liver** | 5.14 | 0.82 | 7 | 0.50 | 0.11 | 7 | 5.93 | 0.72 | 7 |
| **Tumor-to-Lung** | 5.48 | 0.89 | 7 | 0.47 | 0.15 | 7 | 9.95 | 1.99 | 7 |
| **Tumor-to-Spleen** | 10.20 | 1.86 | 7 | 1.14 | 0.43 | 7 | 16.57 | 4.27 | 7 |
| **Tumor-to-Bone** | 35.99 | 10.19 | 7 | 2.67 | 1.06 | 7 | 43.48 | 19.21 | 7 |
